# suPAR remains uninfluenced by surgery in septic patients with bloodstream infection

**DOI:** 10.3205/id000022

**Published:** 2016-07-18

**Authors:** Jasmin Rabensteiner, Florian Prüller, Jürgen Prattes, Thomas Valentin, Ines Zollner-Schwetz, Robert Krause, Martin Hoenigl

**Affiliations:** 1 Clinical Institute of Medical and Chemical Laboratory Diagnostics, Medical University of Graz, Austria; 2 Section of Infectious Diseases and Tropical Medicine, Department of Internal Medicine, Medical University of Graz, Austria; 3 Division of Infectious Diseases, Department of Medicine, University of California, San Diego, CA, USA

**Keywords:** sepsis biomarker, surgical intervention, soluble urokinase plasminogen activator receptor (suPAR), procalcitonin (PCT), interleukin-6 (IL-6), C-reactive protein (CRP)

## Abstract

Surgical trauma induces activation of the immune system and may cause an increase of inflammatory biomarkers tested postoperatively in septic patients treated for bloodstream infection. The aim of this study was to determine the impact of surgical interventions on the novel sepsis biomarker soluble urokinase plasminogen activator receptor (suPAR) and to compare results with those of routine laboratory parameters CRP, PCT, and IL-6 in patients with culture-proven bloodstream infection. Forty-six adult patients with positive blood culture undergoing minor or major surgical intervention were investigated, 12 blood culture positive patients served as control group. Blood was collected 24 hours before and after surgical intervention for determination of the sepsis biomarkers suPAR, CRP, PCT, and IL-6. Within the surgical study cohort, a non-significant increase of suPAR, CRP, and PCT was observed postoperatively (*p* 0.642; *p* 0.773; *p* 0.087). In contrast, a slight decrease of IL-6 (*p* 0.599) was observed. A significant correlation was calculated for the pre- and postoperative difference of CRP (*p* 0.028) and PCT (*p *0.008) and type of surgical intervention received: after minor surgical intervention only PCT decreased significantly (*p*<0.001), while after major surgical interventions no significant differences were observed for all biomarkers evaluated. In the control group, a significant decrease of CRP (*p* 0.005) and PCT (*p* 0.005) was observed. In patients treated adequately for bloodstream infections, postoperative suPAR levels remained uninfluenced of the surgical trauma and might therefore be a reliable parameter for postoperative infectious monitoring. After minor surgical intervention, PCT seems to be the most reliable parameter.

## Introduction

Sepsis is a major health concern associated with significant morbidity and mortality [1], [2], [3]. Different biomarkers are used for diagnosis and therapy monitoring of systemic infections, especially C-reactive protein (CRP), procalcitonin (PCT), and interleukin-6 (IL-6) [4], [5], [6]. Antiinfective therapy monitoring becomes complicated once a patient has to undergo surgical interventions while being treated for bloodstream infection. It is well known that any surgical trauma causes local activation of various white blood cells, which release different cytokines and mediators for further activation of the immune system [7]. Infectious complication is the most important differential diagnosis for a postoperative increase of biomarkers in septic patients. The decision whether or not to adapt/escalate the antiinfective regimen in patients with a postoperative increase of biomarkers or to choose a “wait-and-see” strategy with further monitoring of biomarkers therefore remains a challenge. In patients with increasing sepsis biomarkers after surgery a possible iatrogenic driven elevation of these markers due to the surgical trauma per se may lead to misinterpretation of test results and hence of the clinical patient’s condition.

The aim of this study was to determine the impact of surgical interventions on the novel sepsis biomarker soluble urokinase plasminogen activator receptor (suPAR) [4], [6], [8], [9] and to compare with the routinely used laboratory parameters CRP, PCT, and IL-6 in patients with culture-proven bloodstream infection.

## Methods

This retrospective analysis of a cohort study evaluated patients who were enrolled into the NOBIS study (ClinicalTrials.gov Identifier: NCT01359891) between May 2011 and July 2012 at the University Hospital Graz, Austria. Study samples were collected as part of the participation in the NOBIS study, which enrolled more than 700 patients with bloodstream infection [4], [5], [6]. For participation to the NOBIS study informed consent was required prior to sample collection. The objective of this analysis was to evaluate the impact of surgery on inflammatory biomarkers in patients treated adequately for bloodstream infection.

NOBIS participants with culture-proven bacteremia were included in this analysis if they met the following inclusion criteria: (i) age above 18 years, (ii) presentation at or admitted to the University Hospital of Graz, Austria, (iii) fulfillment of systemic inflammatory response syndrome criteria and clinical suspicion of bacteremia/septicemia by the attending physician, (iv) positive result of drawn blood culture, (v) surgical intervention in the following ten days after initial positive blood culture, (vi) blood sample within 24 hours before and after surgical intervention, (vii) absence of local, organic and systemic infectious complications until seven postoperative days, and (viii) informed consent. 

A total of 58 patients met the inclusion criteria. Patients’ medical records were reviewed by using a standardized data collection template in order to collect demographic information and clinical data. Type of surgical intervention and laboratory test results for CRP, PCT, suPAR and IL-6, as well as microbiological results were extracted from computerized databases. 

Fourty-six patients with positive blood culture result were included and two different groups of surgical interventions were defined: minor and major (Table 1 (Tab. 1)). Minor surgical interventions were classified as invasive interventions with minimal or low tissue damage; and major surgical interventions were defined as operations with high amount of tissue damage, e.g. liver transplantation or open valve repair surgery. Another 12 patients with positive blood culture, and subsequent surgical intervention of implantation or explantation of central venous access served as the control group (Table 1 (Tab. 1)). All 58 patients received adequate antibiotic or antifungal therapy according to the susceptibility testing of the microbiological laboratory. This study was approved by the ethics committee of the Medical University of Graz, Austria (EC-number 21–469 ex 09/10).

Three pairs of blood culture bottles (BACTEC™ Plus Aerobic/F and Anaerobic/F) were drawn from each patient and incubated in the continuously monitored blood culture instrument BD BACTEC™ FX (BD Diagnostics, Sparks, MD). Positive blood cultures were further processed; microbiological identifications of responsible pathogens were performed by routine laboratory measures in the Microbiological Laboratory, Medical University Graz. Detailed results of determined pathogens are displayed in Table 2 (Tab. 2).

Lithium heparin plasma samples were obtained for testing CRP, PCT, and IL-6 immediately after blood collection on a fully automated laboratory analyzer (Cobas 8000 system, Roche Diagnostics, Rotkreuz, Switzerland). Determination of suPAR was performed from EDTA plasma samples which were immediately stored at –80°C after blood collection using the suPARnostic™ ELISA kit (ViroGates, Copenhagen, Denmark) according to the manufactures instructions. Measurements were performed at the Clinical Institute of Medical and Chemical Laboratory Diagnostics, Medical University Graz, on an ELISA platform reader (Flex Station 3, Molecular Devices, Munich, Germany).

Statistical analysis was performed using SPSS, version 21 (SPSS Inc., Chicago, IL, USA). Continuous data (*e.g.* suPAR values) are presented as medians with interquartile ranges (IQR) or means (± standard deviation), and categorical data as proportions. A *p*-value of less than 0.05 was considered statistically significant, using Wilcoxon test. Correlation was calculated using Spearman-Rho calculation analysis. We performed a sample size calculation for the comparison of pre- and postoperative suPAR levels for the two groups undergoing surgery using paired t-test and powered the study for detection of an effect size that corresponds to a standardized effect size of 0.65, assuming normal data. When alpha (two sided) =0.05 and power=0.80, 21 patients need to be included per group, with pre- and postoperative suPAR levels available. The control group was powered for detection of a standardized effect size of 0.9.

## Results

No significant correlation was found between the number of blood culture positive days before surgical intervention (median 3 days, range 0–10) and the difference of pre- and postoperative suPAR, CRP, PCT, and IL-6 levels.

Within the whole study cohort, no significant change of suPAR (*p* 0.642), CRP (*p* 0.773), PCT (*p* 0.087), and IL-6 (*p* 0.599) was observed postoperatively (Table 3 (Tab. 3)). 

In the control group, a significant decrease of CRP (*p* 0.005) and PCT (*p* 0.005) was observed, while suPAR (*p* 0.071) and IL-6 (*p* 0.239) did not decrease significantly (Table 4 (Tab. 4)). In patients receiving minor surgical interventions, only PCT (*p*<0.001) showed a significant postoperative decrease, whereas for suPAR (*p* 0.783), CRP (*p* 0.375), and IL-6 (*p* 0.543) no significant change was calculated (Table 4 (Tab. 4)). In patients with major surgical interventions no postoperative de- or increase of suPAR (*p* 0.407), CRP (*p* 0.530), PCT (*p* 0.209), and IL-6 (*p* 0.855) was observed (Table 4 (Tab. 4)). When differences between the pre- and postoperative levels of the 4 biomarkers suPAR, CRP, PCT, and IL-6 were analyzed according to the 3 different groups of surgical intervention (minor, major, and control), a significant correlation was calculated for CRP (*p* 0.028) and PCT (*p* 0.008).

## Discussion

In this study, the impact of tissue damage due to surgical interventions on the sepsis biomarkers suPAR, CRP, PCT, and IL-6 in patients treated for culture-proven bloodstream infection was investigated. 

In patients with increasing sepsis biomarkers after surgery a possible iatrogenic driven elevation of these markers due to the surgical trauma *per se* may lead to misinterpretation of test results and hence of the clinical patient’s condition. Surgical trauma can induce an increase of CRP and IL-6 levels strongly depending on the size of local tissue damage [10], [11], [12]. Previous studies have reported that IL-6, after reaching the peak immediately after surgical intervention decreases rapidly during the first postoperative day [13]. PCT is mainly stimulated by bacterial endotoxins and might therefore be a more suitable and specific parameter for the early postoperative period [14], [15]. SuPAR has proven to be a useful parameter to predict bacteremia in patients presenting with systemic inflammatory response syndrome [4], [16]. High suPAR levels on the first postoperative day may be therefore of special interest. 

In this study, suPAR, CRP, and IL-6 remained on a steady level in the early postoperative phase in patients receiving minor but also major surgery. These results suggest that suPAR remains uninfluenced by surgical trauma *per se* and therefore seem to be a possible parameter for diagnosing early postoperative infectious complications. Similar results were reported by Gozdzik and colleagues. SuPAR plasma levels remained stable in patients undergoing elective coronary bypass graft surgery under cardiopulmonary bypass. No significant changes were determined either 6 or 24 hours postoperatively [17]. Svendsen et al. also revealed that high concentrations of suPAR are significantly associated with the development of postoperative pneumonia after elective surgery for colorectal cancer [18].

PCT showed a significant postoperative decrease after minor surgical intervention and in the control group. According to the literature, PCT seems to be a useful, independent parameter for detection of infectious postoperative complications, especially at the postoperative day 1 and can also be used for postoperative monitoring for antiinfective therapy [19], [20]. CRP showed a significant decrease only in the control group. Therefore we suggest that especially after minor surgical interventions, PCT is the most reliable parameter for early postoperative infectious complications. After major surgical intervention no parameter of the 4 tested was superior and therefore no suggestion can be made. Since currently suPAR is only used for research use, the remaining three biomarkers CRP, PCT, and IL-6 have to be used in the routine, all showing equal diagnostic potential to suPAR.

This study design also has some limitations, in particular numbers of patients were small for the different types of surgical interventions.

## Conclusions

In conclusion suPAR remained on a steady level in the early postoperative phase and seems not to be influenced by the local tissue damage in patients receiving minor or major surgical intervention. PCT was the most reliable parameter for early postoperative infectious monitoring after minor surgical interventions, while after major surgical intervention none of the four parameters tested was shown to be superior regarding diagnostic potential.

### Key Messages

1. Surgical trauma *per se* may lead to an iatrogenic driven elevation of sepsis biomarkers due to local tissue damage.
2. Postoperative suPAR levels remained uninfluenced irrespective of type of surgery.
3. PCT showed a significant decrease in patients receiving minor surgery and in the control group.

## Abbreviations

- CRP – C-reactive protein
- IL-6 – interleukin-6
- IQR – interquartile ranges
- PCT – procalcitonin
- suPAR – soluble urokinase plasminogen activator receptor

## Notes

### Study registration

ClinicalTrials.gov Identifier: NCT01359891

Registration date: May 23, 2011

### Acknowledgments

The authors thank Christina Strempfl, Bernadette Neuhold, and Verena Posch for their support in the microbiologic laboratory and Elisabeth Winter for performing suPAR assays for discussion of results.

### Conflicts of interest

SuPAR kits used in the study were provided by the companies’ suPARnostic (Kopenhagen, Denmark) and Biomedica Medical Products (Vienna, Austria). No other funding was obtained.

The authors declare no conflict of interest.

## Figures and Tables

**Table 1:**
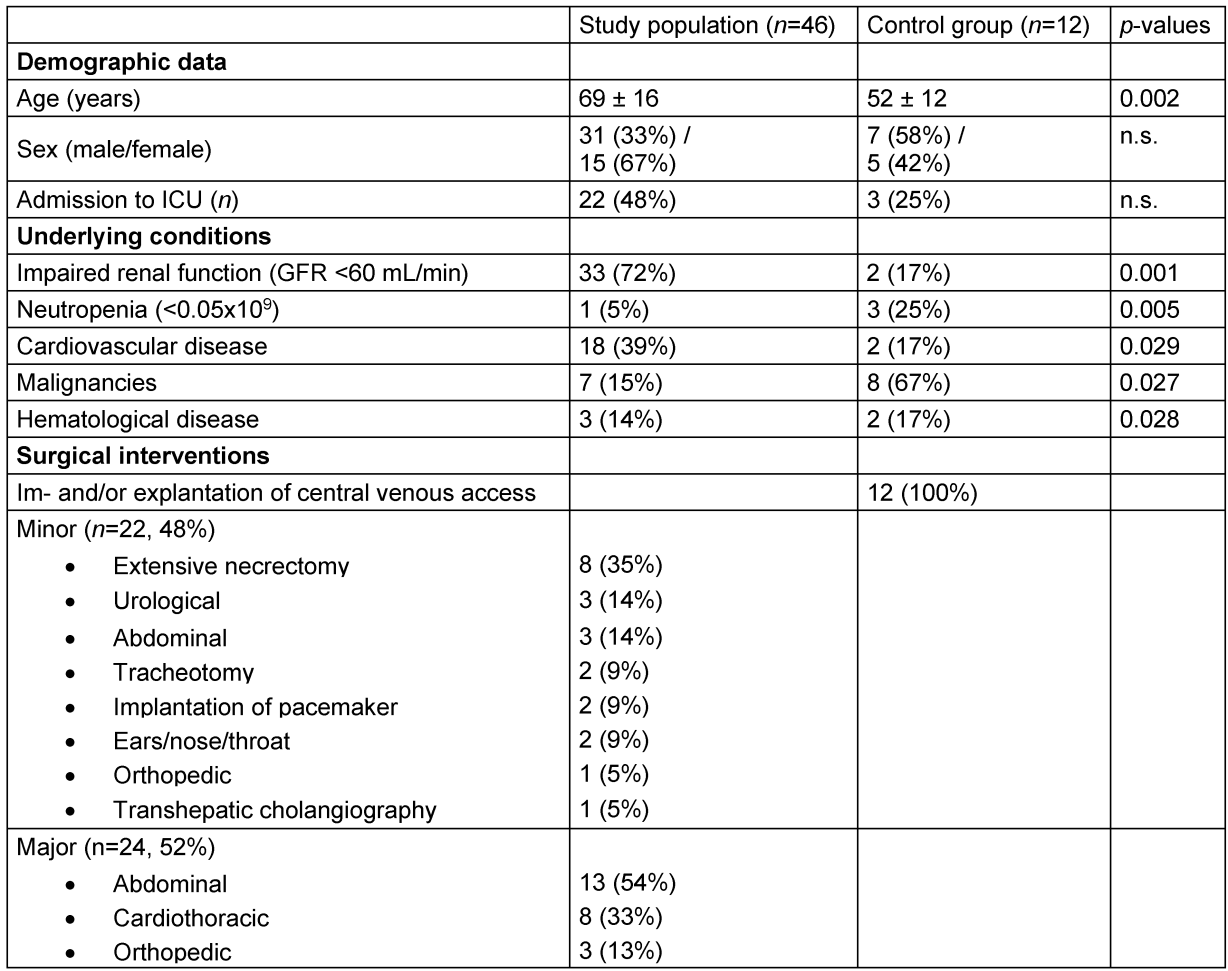
Baseline characteristics of the study cohort (*n*=58). Baseline characteristics (mean ± standard deviation, number *n* and proportion in %), including type of surgical intervention.

**Table 2:**
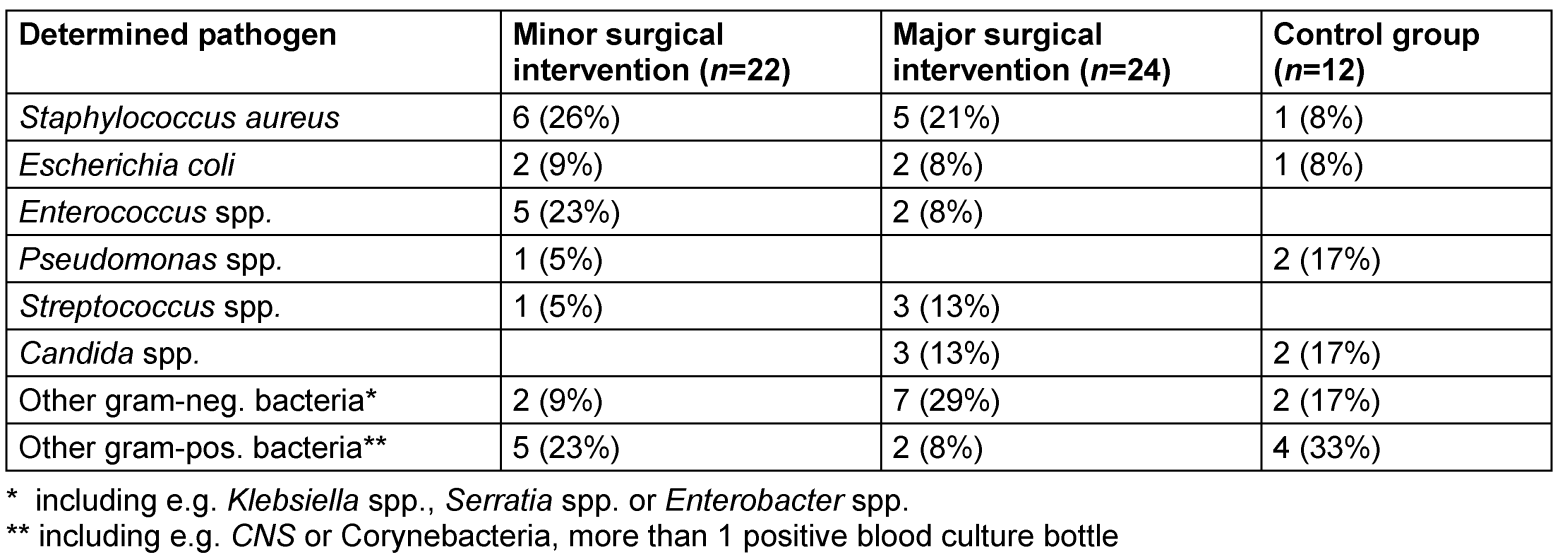
Microbiological spectrum of blood stream infections. Detailed information about bacteria and fungi (number *n* and proportion in %) responsible for bloodstream infection. No significant difference between the groups was found in the microbiological spectrum (*p* 0.241).

**Table 3:**
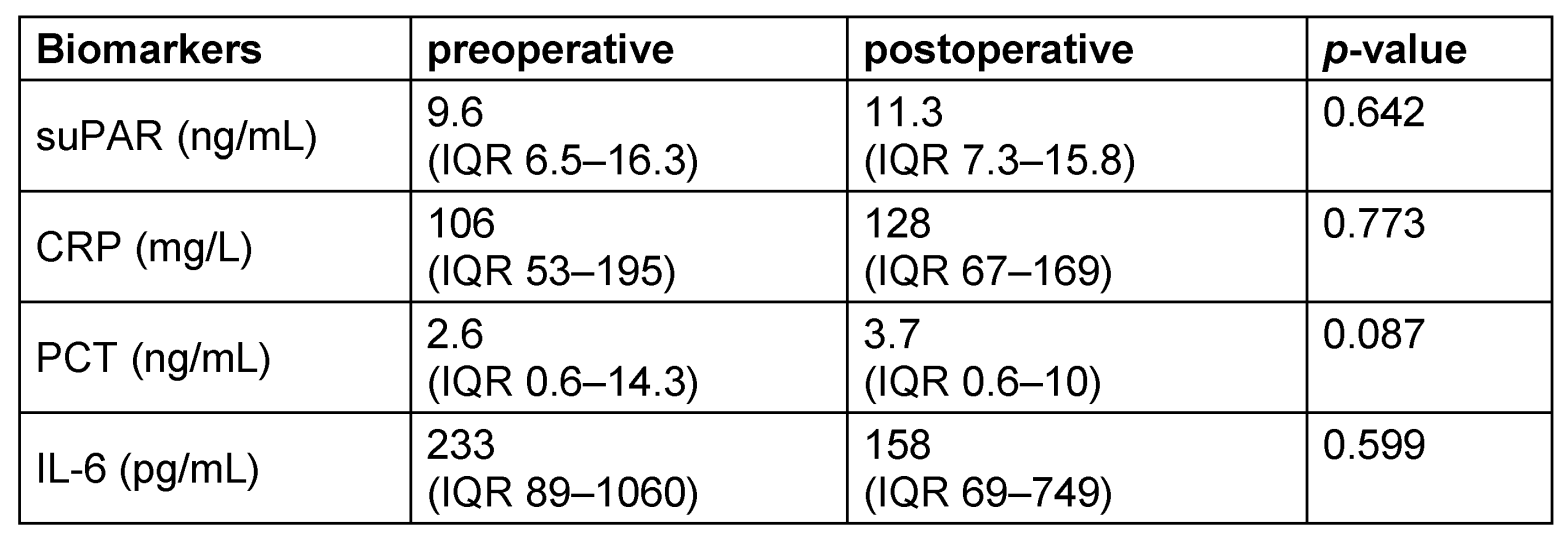
Pre- and postoperative levels of biomarkers. Results of the tested parameters suPAR, CRP, PCT, and IL-6 levels (median and IQR) pre- and postoperative, displayed for the total surgical intervention study cohort (*n*=46).

**Table 4:**
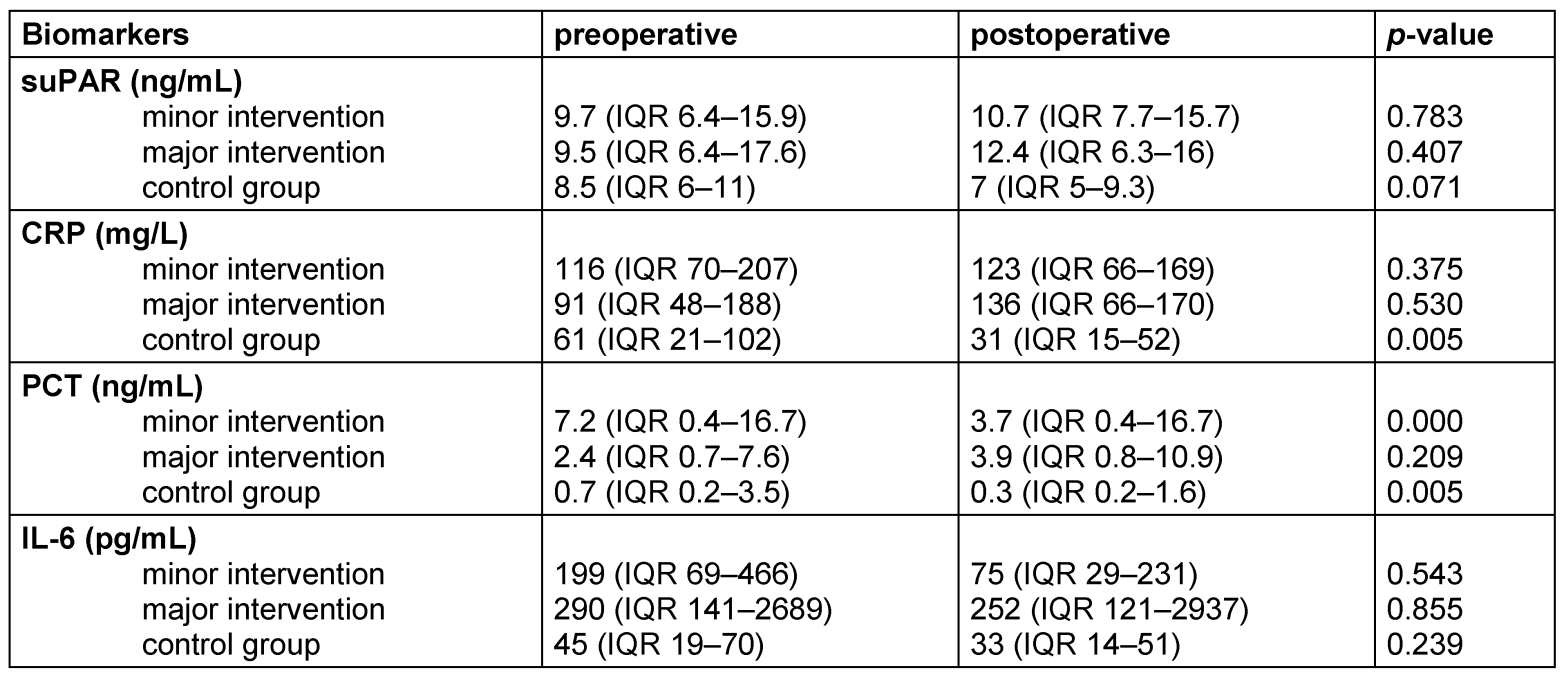
Pre- and postoperative levels of biomarkers based on type of surgical intervention. SuPAR, CRP, PCT, and IL-6 levels (median and IQR) pre- and postoperative and *p*-values displayed for subgroups receiving minor (*n*=22), major surgical interventions (*n*=24) and control group (*n*=12).
